# Cooperative Blood-feeding and the Function and Implications of Feeding Aggregations in the Sand Fly, *Lutzomyia longipalpis* (Diptera: Psychodidae)

**DOI:** 10.1371/journal.pntd.0000503

**Published:** 2009-08-18

**Authors:** Frédéric Tripet, Simon Clegg, Dia-Eldin Elnaiem, Richard D. Ward

**Affiliations:** 1 School of Life Sciences, Keele University, Keele, Staffordshire, United Kingdom; 2 Laboratory of Malaria and Vector Research, National Instutute of Allergy and Infectious Diseases, National Institutes of Health, Maryland, United States of America; Liverpool School of Tropical Medicine, United Kingdom

## Abstract

Given the importance that the evolution of cooperation bears in evolutionary biology and the social sciences, extensive theoretical work has focused on identifying conditions that promote cooperation among individuals. In insects, cooperative or altruistic interactions typically occur amongst social insects and are thus explained by kin selection. Here we provide evidence that in *Lutzomia longipalpis*, a small biting fly and an important vector of leishmaniasis in the New World, cooperative blood-feeding in groups of non-kin individuals results in a strong decrease in saliva expenditure. Feeding in groups also strongly affected the time taken to initiate a bloodmeal and its duration and ultimately resulted in greater fecundity. The benefits of feeding aggregations were particularly strong when flies fed on older hosts pre-exposed to sand fly bites, suggesting that flies feeding in groups may be better able to overcome their stronger immune response. These results demonstrate that, in *L. longipalpis*, feeding cooperatively maximizes the effects of salivary components injected into hosts to facilitate blood intake and to counteract the host immune defences. As a result, cooperating sand flies enjoy enormous fitness gains. This constitutes, to our knowledge, the first functional explanation for feeding aggregations in this species and potentially in other hematophagous insects and a rare example of cooperation amongst individuals of a non-social insects species. The evolution of cooperative group feeding in sand flies may have important implications for the epidemiology of leishmaniasis.

## Author Summary

Understanding the processes that promote cooperation amongst animals in nature is a fundamental question in evolutionary biology with ramifications in the social sciences. In insects, cooperative or altruistic interactions are usually observed amongst genetically related social insects (kin selection). Here we provide evidence that in *Lutzomia longipalpis*, a small biting fly and an important vector of disease in the New World, cooperative blood-feeding occurs in groups of non-kin individuals. Groups of 20 flies and single flies were fed on hamster hosts and we compared their salivary gland usage as well as the time taken to initiate a bloodmeal, its duration, and the number of eggs they produced. Our results show that flies feeding in aggregations benefit from decreased saliva expenditure and greatly enhanced blood intake and egg production. These effects were particularly strong on older hamsters pre-exposed to sand fly bites, suggesting that group-feeding flies may better overcome their stronger immune response. These experiments demonstrate that, in *L. longipalpis*, feeding cooperatively maximizes the effects of saliva injected into hosts to facilitate blood intake and to counteract the host immune defences, resulting in much increased fecundity. This constitutes the first explanation for the function of feeding aggregations in hematophagous insects and a fascinating example of cooperation amongst individuals in a non-social organism.

## Introduction

**Figure 1:**
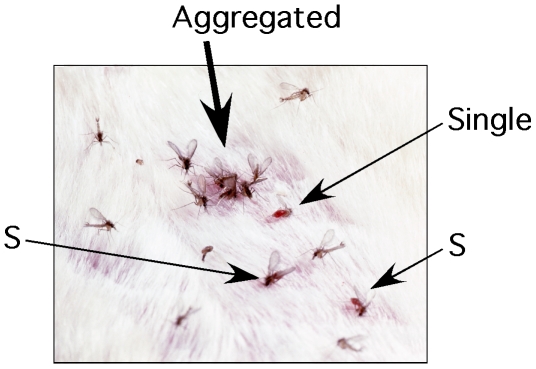
Aggregated and single female flies feeding on a vertebrate host with males in courtship display in the vicinity of feeding females.

Phlebotomine sand flies (Diptera: Psychodidae) are important vectors of leishmaniasis, a parasitic disease affecting an estimated 1–1.5 millions people and causing over 50'000 deaths worldwide each year [1]. Medical entomologists have long noticed that some sand fly species tend to arrive in waves to bite mammal hosts, that they feed in aggregations and that new-arriving flies promptly join existing groups [2]. Feeding aggregations have also been observed in the laboratory, including in the New World sand fly *Lutzomyia longipalpis* which is commonly reared for research on drugs, vaccines and chemical attractants (Fig. 1), The functional explanation of this ‘invitational effect’ [3], which in *L. longipalpis* has been shown to be mediated by a pheromone produced on the female maxillary palps [4] so far has eluded scientists.

Because sand flies are tiny (2–4 mm) and produce large amounts of potent vasodilators [5],[6] to facilitate blood intake, we hypothesized that individual flies might benefit from inviting other flies to feed in their vicinity by sparing costly saliva. The ‘cooperative feeding hypothesis’ would provide a simple explanation for the invitational effect observed in group-feeding sand fly species and, possibly, in a number of other group-feeding hematophagous dipteran species [4],[7],[8],[9].

This hypothesis is also intriguing from an evolutionary point of view because cooperative interactions in insects are usually seen in social insects where it occurs among related individuals and can thus be explained through kin selection [10],[11]. Given that sand flies, as most Diptera, do not provide parental care to their offspring [12] and are known to disperse over substantial distances in search of hosts [13],[14], cooperative feeding occurs amongst unrelated flies and would constitute a rare example of non-kin cooperation in insects.

Finally, understanding the dynamics and function of feeding aggregations in sand flies and its consequences in terms of saliva usage bears special significance as salivary components have been implicated in the successful development of *Leishmania* parasites in the host [6],[15]. In Old and New world sand fly genera *Phlebomomus* and *Lutzomiya*, co-injecting salivary gland extracts with *Leishmania* parasite enhances infection, which translates in increased lesion size at the site of the bite and higher parasite burden within those lesions [16],[17],[18],[19],[20],[21]. This suggests that a behavioural trait such as cooperative feeding, which results in saliva being injected by multiple flies at a single biting site on the host, could potentially also play an important role in the dynamics and severity of *Leishmania* infections.

We tested the cooperative feeding hypothesis in *L. longipalpis*, an important New-World vector of visceral leishmaniasis. Sand flies were blood-fed on a host either in groups or singly and the effect of group-feeding on their salivary use, feeding duration and fecundity was recorded. The results show that *L. longipalpis* individuals prefer to feed in aggregations and that by doing so greatly increase feeding efficiency, bloomeal profitability and fecundity. The implications of these findings for our understanding of the evolution and maintenance of cooperation in non-social organisms and for the epidemiology of leishmaniasis are discussed.

## Materials and Methods

### Experimental system

All animal handling and anaesthetizing procedures used in these experiments were approved by the Keele University sub-ethics committee and carried on under the conditions specified by the UK Home Office Animals Scientific Procedures Act. Experimental designs aimed at minimizing the number of hamsters used while achieving adequate statistical power. Two groups of golden hamsters, respectively 3-months and 12-months old were used in this study. Three-months old hamsters were naïve to sand fly bites whilst the 12-months old ones had been exposed to *Lutzomyia* sand fly bites on average once every 1–1.5 months prior to our experiments. At the time of the experiment they had not been exposed to bites for a period of 30 days. The *L. longipalpis s.l.* colony used in all experiments (except some preliminary observations – see below) was established in the 1980's from a large number of wild-caught females collected in Jacobina (11° 11′S, 40° 30′W), which is located in Bahia State, Brazil. This population is characterized by the 3M∝H, sesquiterpene 3-methyl-∝-himachalene male sex pheromone type as well as a characteristic mating song and may thus represent a distinct sub-species of *L. longipalpis* [22],[23]. All flies were used 4 d after emergence and given sugar water until blood-feeding. Flies were picked at random but males were excluded from all experiments in order to avoid confounding effects of male-produced courtship pheromones on female feeding aggregations.

### Preliminary feeding aggregation tests

Before initiating the study, we established that sand flies kept in our laboratory colonies showed the same inclination for feeding in aggregations as what has been reported for their wild counterparts. To do so, forty female flies were released in a cage with a sedated hamster covered with a slotted paper-towel so as to expose its 4 paws. The distribution of feeding sand flies on the paws was recorded at 1 min intervals. For the time interval at which the highest number of flies was feeding and in 3 replicates, a chi-squared test was conducted on the number of flies feeding per paw to reveal significant deviations from random distribution. Significant aggregation was observed in two of the tests and flies tended to aggregate in the third replicate (Likelihood-ratio: *X* ^2^ = 5.7, *n* = 17, *P* = 0.002), (*X* ^2^ = 6.3, *n* = 17, *P* = 0.097) and (*X* ^2^ = 13.6, *n* = 18, *P* = 0.004), yielding a combined *p*-value<0.001. Feeding aggregation was also observed amongst a mixture of 20 individuals from two *L. longipalpis* colonies from Marajo, district of Pará, Brazil and El Callejon in Colombia. These populations are characterized by the cembrene-1 and 9MGB, sesquiterpene (S) 9-methyl-germacrene B pheromonal types and contrasting mating songs suggesting that they may also represent distinct sibling species of *L. longipalpis* [22],[23].

### Measures of saliva expenditure

Groups of 20 female flies were fed on a single exposed hamster paw. For single flies, 5 flies were introduced in the cage and when the first fly initiated feeding we carefully aspirated the remaining flies. Five replicates of group feeding alternating with single-fly feeds were conducted on 5 young hamsters (3 months-old). After each experiment 6 randomly picked group-fed flies and all single-fed flies were dissected in a saline solution and a digital picture of the salivary gland pair was taken. The surface of the salivary gland pictures was measured on screen using the programme ImageJ1.38 available at http://rsb.info.nih.gov/nih-image/. All measurements were conducted blind with regard to the flies' original groups. The data was analyzed non-parametrically to account for non-normality.

### Measures of feeding duration and egg production

A design similar to that described above was used but in group-fed flies we first powder-dyed 10 of the 20 flies to facilitate behavioural observations. Group and single-fed flies were either fed on a young naïve or an older exposed hamster (see details above) to study the potential effect of an enhanced immune response due to previous exposures to sand fly bites. As above and to avoid biases, 4 hamsters (replicates) were used alternately to feed groups of flies or single flies. The time at which flies initiated and finished blood intake was recorded and the first 6–10 individuals that initiated feeding where captured with an aspirator as they completed their blood meal and left the host. Group and single-fed flies were set to lay eggs singly in a vial containing a damp filter paper folded to produce a concertina effect.

All data were checked for deviation from normality, unequal variances and heteroscedasticity and analyzed using JMP6.0 available at http://www.jmp.com/.

## Results

### Bloodmeal initiation

**Figure 2:**
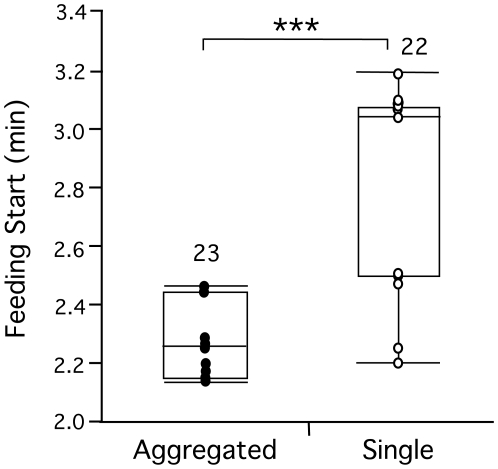
The time taken to initiate a bloodmeal in flies feeding in groups. Data points are shown and quartiles and sample sizes are indicated. *P*-values are *P*<0.001 ***.

In line with the cooperation hypothesis, single flies presented a host were so reluctant to feed that we had to give them temporary companion flies to initiate blood-feeding. In contrast, group-feeding flies started feeding significantly faster (Mann-Whitney: *Z* = 4.56, *n* = 45, *P*<0.001) (Table 1). The variance in the time at which blood-feeding started was also an order of magnitude smaller in group-feeding flies than in single-feeding flies indicating that they fed synchronously (Bartlett: *F*_1, 44_ = 21.5, *P*<0.001) (Fig. 2). Since older hosts that have been more exposed to bites may exhibit a stronger immune response to bites or tougher skin, we also compared the time it took to initiate the blood meal in relation to host age. Given the unequal variances this was done non-parametrically across both experimental groups. There was no effect of host age on the time taken by flies to initiate their bloodmeal (Mann-Whitney: *Z* = 0.09, *n* = 45, *p* = 0.927).

**Table 1:** The time taken to initiate a bloodmeal (min), its duration (min), the resulting number of eggs produced and profitability measured as the number of eggs produced per unit of time spent feeding (min) in flies feeding singly or in groups (20 individuals) on naive and older pre-exposed hamsters.

Aggregation level	Host	Sample size (n)	Bloodmeal initiation (min)	Bloodmeal duration (min)	No Eggs	Profitability (eggs/feeding duration)
**Single fly**	**naive**	13	2.8±0.3	7.4±3.2	29.5±9.7	4.7±3.3
	**pre-exposed**	9	2.7±0.4	23.6±8.5	39.6±7.7	2.1±1.6
**Group of flies**	**naive**	12	2.2±0.1	7.2±3.5	65.3±10.4	11.8±6.6
	**Pre-exposed**	11	2.4±0.1	9.5±1.5	70.8±10.9	7.7±2.0

All data are means±SD (sample size).

### Feeding duration

**Figure 3:**
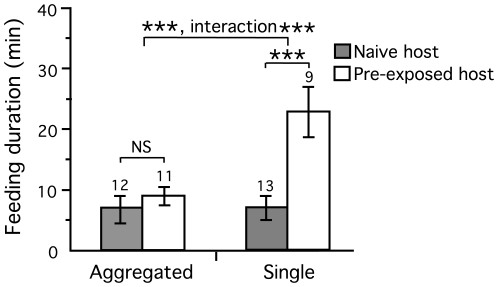
Difference in feeding duration in flies feeding on older pre-exposed hosts (white boxes) or on young naïve hosts (grey boxes) in groups or singly. Data are means±SEM and sample sizes are indicated. Significance levels are shown for comparisons between flies fed on naive and pre-exposed hosts within experimental groups, for the effect of aggregation and for the interaction between aggregation level and host age (interaction). *P*-values are *P*<0.001 ***, non-significant NS.

**Figure 4:**
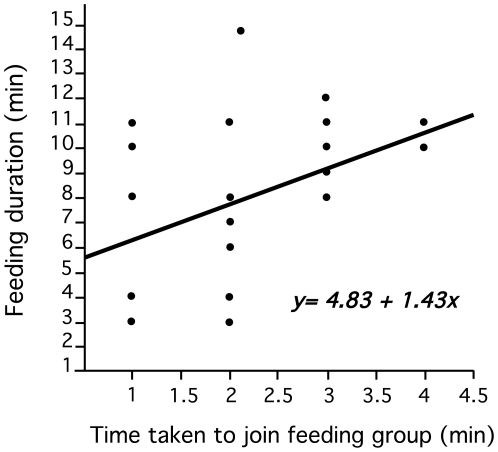
Relationship between the time taken to join the aggregation and the duration of feeding (min) in group-feeding sand flies.

Group feeding strongly decreased the time taken to acquire a bloodmeal. Overall single-feeding flies spend 70.5% longer acquiring their bloodmeal than group-feeding ones. Host age also had a very strong effect on feeding duration. Single flies took much longer to feed, but particularly so when feeding on older pre-exposed hosts (2-way Anova: aggregation, *T*_1,41_ = −5.2, *p*<0.001; host age, *T*_1,41_ = −6.7, *P*<0.001; interaction, *T*_1,41_ = 5.0, *P*<0.001; *r*-square = 0.674) (Table 1, Fig. 3). Feeding on an older hamster resulted in a 219% increase in feeding time in this group, whilst in group-feeding flies feeding on young naïve hamsters, the duration increased by 31.9% only. Importantly, in group-feeding flies the order in which the flies initiated feeding had a significant effect on the duration of their blood meal. The later a fly joined a group and/or initiated feeding, the longer it took her to acquire a bloodmeal suggesting that there maybe a cost in delaying feeding or waiting for other flies to start feeding in cooperating groups (regression: *F*_1,22_ = 7.12, *P* = 0.014) (Fig. 4).

### Saliva expenditure

**Figure 5:**
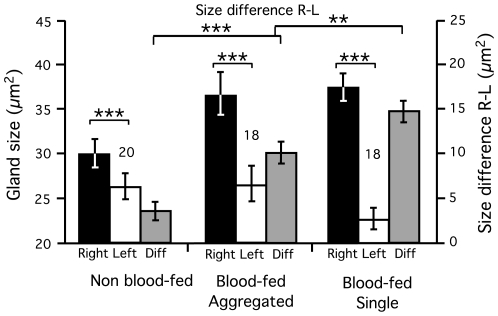
Size of the right (black boxes) and left (white boxes) salivary glands (µm^2^, left scale) as well as the size difference between them (µm^2^, right scale) in flies kept unfed (sugar water only) or feeding singly and in groups on a hamster. Data are means±SEM and sample sizes are indicated. Significance levels are shown for mean differences between the right and left glands (difference L-R) between experimental groups and for comparisons between left and right glands (paired within individuals) within each group (see results for details). *P*-values are *P*<0.001 ***, *P*<0.01 **.

Using digital pictures of dissected salivary gland pairs and imaging software (see methods), we compared salivary gland use for a single bloodmeal in single and group-feeding flies as well as in unfed flies. All blood-fed flies were fed on young hamsters in this experiment. After blood-feeding, flies kept in groups or singly had significantly larger right glands than flies that were kept unfed suggesting that flies produced saliva whilst feeding instead of simply emptying existing reserves (Kruskal-Wallis: *X* ^2^ = 10.2, n = 56, *P*<0.006). In contrast, in all groups the left gland was significantly smaller than the right gland (Wilcoxon Sign-Rank: *P*<0.001 in all cases). The difference between the two glands was significantly smaller in unfed flies than in both groups of blood-fed flies suggesting that they produce but also use more saliva (Mann-Whitney: *P*<0.001 in both cases) (Table 2 and Fig. 5). There was no statistical difference between the right gland of flies fed on hamsters in groups or singly. However aggregation had a strong effect on the difference in size between the right and left glands, with single flies having an average 45% less saliva in their left salivary gland than those feeding in groups (Mann-Whitney: *X* ^2^ = 20.9, *n* = 36, *P* = 0.004) (Table 2 and Fig. 5). This comparison was particularly statistically powerful as it effectively controlled the data for variation in body size (power equal to 1); something we could not achieve by controlling gland size by wing length because the two variables did not correlate (Pearson correlation: *P*>0.05 in all three groups).

**Table 2:** Size of the right and left glands (µm^2^) and the size difference between them (µm^2^) in flies kept unfed (with sugar water available) and flies fed singly or in groups (20 individuals) on a naive hamster.

Experimental group	Sample size (n)	Right gland size (µm^2^)	Left gland size (µm^2^)	Size difference between glands (µm^2^)
**Unfed flies**	20	29.9±7.8	26.3±7.2	3.6±2.5
**Single blood-fed fly**	18	37.6±6.2	22.8±5.3	14.8±4.9
**Group blood-fed flies**	18	36.9±9.6	26.7±9.2	10.2±6.1

All data are means±SD (sample size).

### Egg production

**Figure 6:**
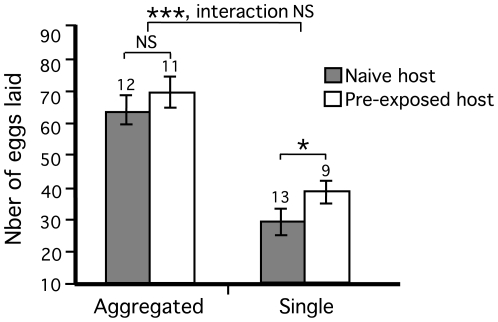
Fecundity measured as the number of eggs laid following a bloodmeal. Data are means±SEM and sample sizes are indicated. *P*-values are *P*<0.001 ***, *P*<0.05 *, non-significant NS.

**Figure 7:**
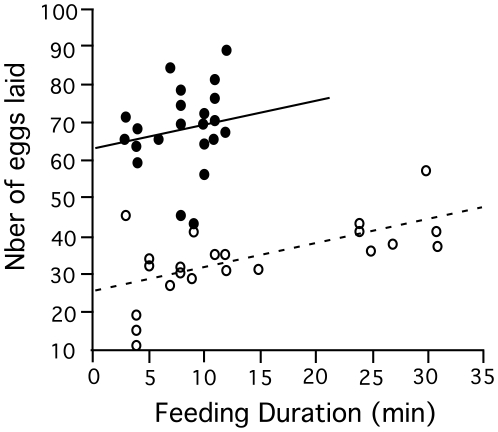
Fecundity measured as the number of eggs laid after a bloodmeal in flies feeding singly (white dots) and in groups (black dots) in relation to feeding duration (min).

The number of eggs laid by females following a bloodmeal was dramatically reduced in flies feeding singly (50.5% decrease). Fecundity was also significantly affected by host age although to a much lesser extend than feeding duration (2-way Anova: aggregation, *T*_1,41_ = 11.3, *P*<0.001; host age, *T*_1,41_ = −2.6, *P* = 0.013; interaction, *T*_1,41_ = 0.8, NS, *r*-square = 0.778) (Fig. 6). Despite a non-significant interaction term in the latter analysis, the data suggest a much stronger effect of host age on fecundity in single-feeding flies than in aggregated flies (Table 1). Indeed, separate analyses of egg production for each experimental treatment revealed no significant difference between flies feeding in groups on younger and older hosts (*T*-test: df = 20, *T* = 1.2, *P* = 0.232) whilst single flies were strongly affected (*T*-test: df = 20, *T* = 2.7, *P* = 0.014). In terms of profitability, single flies that fed on older exposed hosts produced on average 54.4% eggs per unit of time spent feeding than those feeding on young naive hosts (Kruskal-Wallis: *n* = 21, *X* ^2^ = 10.1, *P* = 0.002) (Table 1). Compared to that of group-feeding flies, the profitability of singly-feeding flies on old hosts was 72.9% lower than that of group feeding flies on older hosts and 82.2% lower than those feeding on young hosts (Table 1). The latter two groups did not differ significantly (Kruskal-Wallis: *n* = 23, *X* ^2^ = 1.2, *P* = 0.268). There was a strong linear relationship between feeding duration and the number of eggs laid in both experimental groups (GLM: aggregation, *T*_1,42_ = 12.5, *P*<0.001; feeding duration, *T*_1,42_ = 3.2, *P*<0.001; *r*-square = 0.790) (Fig. 7). Importantly, in group-feeding flies the order in which the flies initiated feeding had a no significant effect on fecundity suggesting that there is no fecundity advantage in delaying feeding or waiting for other flies to start feeding in cooperating groups (regression: *F*_1,22_ = 0.02, *P* = 0.903).

## Discussion

### Fitness benefits of cooperative feeding

Our results show unambiguously that the invitational effect observed in *L. longipalpis* is driven by the enormous benefits of feeding in aggregations over feeding singly. Sand flies are tiny flies and whereas the majority of other blood-feeding insects species rely on long piercing mouthparts for reaching into capillary vessels, sand flies use instead chisel-like mouthparts to cut and pierce the epidermis and dermis of their host in order to create a small hemorrhagic pool upon which they feed [24]. The injection of large amounts of their saliva, rich in anticoagulants, antiplatelets, vasodilators and immunomodulators in the wound, enables them to maintain a constant blood flow throughout their bloodmeal [5],[25]. Here we show that, in *L. longipalpis*, feeding in group not only significantly reduces the amount of saliva used per individual during blood-feeding but also drastically decreases the amount of time taken to acquire their bloodmeal and sharply increases their fecundity. At present the costs of producing pheromones such as that responsible for sand fly feeding aggregations [7] is not known but given our results we can only assume that they are small or largely out-weighed by benefits. Our observations and the results of our experiments suggest that several mechanisms could lead to the fitness gains observed in group-feeding flies. Firstly, individual flies injecting saliva into the host do not only facilitate their own bloodmeal but also that of flies feeding next to them. This result may not be that surprising given that in humans and other mammals *L. longipalpis* bites often cause large erythemas suggesting that the effects of saliva are not narrowly localized [26],[27]. The maxadilan protein alone, an extremely potent vasodilator and an important component of their salivary repertoire, has been shown to elicit such large erythemas both in humans and rabbits [27],[28]. Thus, because the effects of saliva are not narrowly localized, group-feeding flies benefit from the combined anticoagulant, antiplatelet, vasodilatory and immunomodulatory effects of multiple bites and thus produce and spend less saliva acquiring their bloodmeal. As a consequence, they should be able to replenish their salivary glands at a faster rate and could invest more resources in egg production or body maintenance. At present we do not know why flies used saliva from their left gland preferentially. That a paired organ such as salivary glands is used asymmetrically seems counter-intuitive. In contrast to laboratory flies that are fed once, lay eggs, and usually die within their egg-laying pot, wild flies may have more opportunities to feed several times before ovipositing and may use the content of the right gland on those occasions.

Another important source of fitness gain stems from the fact that shortened feeding duration may translate in increased survival. It is generally accepted that host behavioural defences are an important determinant of survival or feeding success in blood-feeding arthropods [29],[30],[31],[32]. Thus, acquiring a bloodmeal faster could decrease the likelihood of eliciting potentially life-threatening host behavioural defences. Albeit feeding in groups may itself entail risks due to density-dependent host defences [30],[33],[34], the much higher blood intake observed in group-feeding flies may overall still translate into better survival, hence higher reproductive value.

Finally, group-feeding *L. longipalpis* females have much higher fecundity despite shortened feeding durations, which suggests that in addition to sparing saliva and feeding more efficiently, they could acquire larger bloodmeals and/or bloodmeals that are easier to digest. Group-feeding flies might not only overwhelm vasoconstriction factors but may also better counteract blood coagulation and immunity factors that would otherwise interfere with bloodmeal acquisition and digestion. Indeed, the much higher feeding durations and lower profitability observed in single flies feeding on older hosts that had been regularly exposed to bites further suggest that single flies may be adversely affected by increased host immune defences. The fact that the overall profitability of group-feeding flies was much higher than that of singly-feeding flies but that those fed on exposed hosts tended to produce less eggs per unit of time spent feeding than those fed on naïve hosts lends further support to that hypothesis. Milleron et al. [35] showed that mice sensitised by sand fly bites or by injection with the maxadilan protein, produced antibodies that reduced vasodilation and negatively affected egg production in *L. longipalpis*. Blood meal size was found to decrease by 10.16% in flies feeding on pre-exposed hosts whilst the number of eggs they produced diminished by 39.5% [35]. Taken together these results emphasize the impact of host immune defence on blood digestion and the fact that flies feeding cooperatively may be better able to counteract them.

In addition to being able to locally inject a combined amount of saliva that is much larger than that of single flies, groups of flies probably inject saliva with a much higher antigenic diversity, and there is some evidence that this could be maximizing its effectiveness [35],[36],[37]. The maxadilan protein is highly polymorphic within and among sand fly populations and between sibling species [36]. Rabbits immunized with different maxadilan variants have been shown to develop antibodies specific to these variants [37]. The same variants bound to the antibodies contained in the serum of individual pigs and humans exposed to sand fly bites in a specific manner [37]. Thus, group-feeding flies may be better able to swamp the host antibody repertoire thereby increasing feeding efficiency and bloodmeal profitability.

### Evolution and maintenance of cooperative feeding

An abundant body of theoretical work in evolutionary ecology has focused on delineating conditions required for the evolution of altruism and cooperation amongst organisms [10],[11]. When kin selection is not implicated, cooperation can evolve in the forms of reciprocity amongst individual that can recognize each other in repeated interactions, or alternatively, as what is know as weak altruism i.e. cooperative interactions amongst individuals that directly benefit from them [10],[11]. Pheromone-mediated aggregations are not uncommon in insects, but only in rare cases have been directly associated with group-feeding benefits in the form of increased resource acquisition [38]. The cooperative interactions observed amongst sand flies are unique in that they do not involve kin selection and their dynamism and high efficiency is reminiscent of those observed in social animals and complex animal societies. Despite the common occurrence of feeding aggregations in nature, which we assume to be consisting largely of unrelated individuals, we were cautious about working on potentially inbred colony material. Our random picking of individuals from large pools of individuals insured that we were working with non-kin individuals but some relatedness would be expected in such an old colony. Consequently, in a preliminary experiment, we tested whether flies belonging to two different cryptic taxa of *L. longipalpis* would feed in ‘mixed’ aggregations. This was observed, proving that cooperative interactions can occur among totally unrelated flies in the laboratory and that they are likely to be found across taxa in mixed sand fly populations in the field.

Cooperative behaviour can only evolve if it is ‘resistant’ to potential cheating individuals. Given that group-feeding flies decrease their salivary expenditure whilst nevertheless feeding faster, it is probable that the increase in feeding efficiency derived from co-injecting larger amounts of saliva into the host levels off at some point. Thus a cheating fly joining a group of flies that already injected their saliva into the host could potentially inject less saliva than other group members and nevertheless benefit from increased blood intake. Although we do not have data on the exact amount of saliva produced and injected by flies in relation to the order in which they initiate feeding in a group, the positive relationship found between feeding order and bloodmeal duration suggest that late-feeding flies may be disadvantaged. Furthermore, early-feeding flies were as fecund as those feeding later thereby confirming the apparent lack of benefits for potentially cheating flies. Thus the complex mode of action of salivary components, their importance for bloodmeal ingestion and their potential role in bloodmeal digestion may prevent cheating, making cooperative feeding an evolutionary stable strategy.

Feeding aggregations have also been observed in other blood-feeding Dipteran species. McCall and Lemon [9] reported an invitational effect in black flies *Simulium damnosum* and the same patterns has been observed in the mosquitoes *Aedes sierrensis* [7], *Ae, aegypti* [7], *Ae. cantans* and others [8]. It is unclear at this point whether the invitational effect observed in these other blood-feeding Dipteran species is symptomatic of similar cooperative processes or if group feeding has a different function in those species.

### Feeding aggregations and *Leishmania* epidemiology

Salivary components are key to the development of *Leismania* parasites inside the host [6],[15]. In both *L. longipalpis* and *L. whitmani*, another important vector species, co-injecting salivary gland extracts with *Leishmania* parasites enhanced the infection, which translated in increased lesion size at the site of the bite and higher parasite burden within those lesions [16],[17],[18],[19],[21]. Understanding how sand fly salivary components, the *Leishmania* parasite and the host immune response interact to determine the course of infections is crucial for the development of vaccines and/or drugs against the disease. It is probably also key to understanding why some human infections develop into the more severe visceral form and why that form is more abundant in certain geographical areas than others [39],[40]. It has also been shown that *Leishmania* actively manipulates sand fly feeding behaviour through the secretion of a promastigote secretory gel (PSG) rich in filamentous proteophosphoglycan (fPPG) that is regurgitated alongside parasites when feeding [41],[42]. Importantly, the PSG and particularly its fPPG component are strong parasite virulence factors [41],[42]. Given the known importance of saliva and PSG for parasite development and the large density-dependent effects we report here on sand fly fitness, there is a possibility that fly density at the bite site could have an impact on the course of an infection. This work therefore begs for follow up studies aimed at exploring the interactive effects of salivary component polymorphism, PSG, and sand fly species composition and density, on disease transmission and pathology.
